# Quality of vitamin K antagonist control and outcomes in atrial fibrillation patients: a meta-analysis and meta-regression

**DOI:** 10.1186/1477-9560-12-14

**Published:** 2014-06-24

**Authors:** Elizabeth S Mearns, C Michael White, Christine G Kohn, Jessica Hawthorne, Ju-Sung Song, Joy Meng, Jeff R Schein, Monika K Raut, Craig I Coleman

**Affiliations:** 1 Department of Pharmacy Practice, University of Connecticut School of Pharmacy, 69 N Eagleville Road, Storrs, CT 06269-3092, USA; 2 Hartford Hospital Division of Cardiology, 80 Seymour Street, Hartford, CT 06102-5037, USA; 3 Janssen Scientific Affairs, LLC, Raritan, NJ, USA

**Keywords:** Vitamin K antagonists, Atrial fibrillation, International normalized ratio, Anticoagulation

## Abstract

**Background:**

Atrial fibrillation (AF) patients frequently require anticoagulation with vitamin K antagonists (VKAs) to prevent thromboembolic events, but their use increases the risk of hemorrhage. We evaluated time spent in therapeutic range (TTR), proportion of international normalized ratio (INR) measurements in range (PINRR), adverse events in relation to INR, and predictors of INR control in AF patients using VKAs.

**Methods:**

We searched MEDLINE, CENTRAL and EMBASE (1990-June 2013) for studies of AF patients receiving adjusted-dose VKAs that reported INR control measures (TTR and PINRR) and/or reported an INR measurement coinciding with thromboembolic or hemorrhagic events. Random-effects meta-analyses and meta-regression were performed.

**Results:**

Ninety-five articles were included. Sixty-eight VKA-treated study groups reported measures of INR control, while 43 studies reported an INR around the time of the adverse event. Patients spent 61% (95% CI, 59–62%), 25% (95% CI, 23–27%) and 14% (95% CI, 13-15%) of their time within, below or above the therapeutic range. PINRR assessments were within, below, and above range 56% (95% CI, 53–59%), 26% (95% CI, 23–29%) and 13% (95% CI, 11-17%) of the time. Patients receiving VKA management in the community spent less TTR than those managed by anticoagulation clinics or in randomized trials. Patients newly receiving VKAs spent less TTR than those with prior VKA use. Patients in Europe/United Kingdom spent more TTR than patients in North America. Fifty-seven percent (95% CI, 50-64%) of thromboembolic events and 42% (95% CI, 35 – 51%) of hemorrhagic events occurred at an INR <2.0 and >3.0, respectively; while 56% (95% CI, 48-64%) of ischemic strokes and 45% of intracranial hemorrhages (95% CI, 29-63%) occurred at INRs <2.0 and >3.0, respectively.

**Conclusions:**

Patients on VKAs for AF frequently have INRs outside the therapeutic range. While, thromboembolic and hemorrhagic events do occur patients with a therapeutic INR; patients with an INR <2.0 make up many of the cases of thromboembolism, while those >3.0 make up many of the cases of hemorrhage. Managing anticoagulation outside of a clinical trial or anticoagulation clinic is associated with poorer INR control, as is, the initiation of therapy in the VKA-naïve. Patients in Europe/UK have better INR control than those in North America.

## Background

Atrial fibrillation (AF) is the most common sustained cardiac arrhythmia worldwide and increases the risk of ischemic stroke by nearly 5-fold [1,2]. Studies have demonstrated that adjusted-dose vitamin K antagonists (VKAs) significantly decrease the risk of stroke in AF patients versus placebo or aspirin therapy [3,4]. However, VKA use can be challenging given the narrow therapeutic international normalized ratio (INR) range, requirement for periodic INR monitoring, high inter-patient variability in response, numerous drug and food interactions and risks related to non-adherence [5].

In previous meta-analyses [6-9] the quality of anticoagulation control with VKAs has proven to be poor; with an estimated time spent in the therapeutic INR range (TTR) between 55% and 64%. Numerous study-level factors (e.g., VKA dosing setting) have been shown to be an important determinant of the quality of INR control. Thromboembolic events may occur more frequently at an INR <2.0 and major hemorrhagic events at an INR >3.0 [10]. No previous meta-analyses have examined INR control in VKA-experienced as compared to naïve patients as a study-level factor, not all analyses have looked at AF alone, and few have assessed the percentage of INRs in therapeutic range (PINRR) as a quality measure of INR control. Moreover, there has been a substantive increase in the number of studies assessing INRs in patients with AF receiving VKAs in the past few years, lending more power and validity to a systematic assessment of INR control being conducted now.

The primary objective of this systematic review with meta-analyses and meta-regression analyses was to assess VKA INR control in AF patients and the effect of selected study-level factors (including prior VKA treatment experience) on TTR and the PINRR, and to evaluate the relationship between the proportion of VKA-associated hemorrhagic and thromboembolic events that occurred when the INR was above or below the therapeutic range.

## Methods

### Study selection

A systematic review of MEDLINE, CENTRAL and EMBASE (from 1990 through June 2013) was performed. Our search strategy for Medline is provided in the Additional file 1. Two investigators reviewed all potentially relevant articles independently, with disagreement resolved by a third investigator. Studies were selected for inclusion on the basis of the following criteria: English full-text randomized controlled trials (RCT), prospective cohort studies or retrospective analyses; contained ≥50 patients in each treatment group; conducted in adult patients (≥18 years of age) receiving dose-adjusted VKA with AF as their primary reason for anticoagulation; and INR control reported as TTR, PINRR or report an INR measurement at (within 48 hours of the event) or near (more than 48 hours from the event) the time of a hemorrhagic or thromboembolic event. Studies were excluded if the duration of study was <3 months, the target INR range was other than 2.0 to 3.0, or patient population overlapped with another study. Manual backward citation tracking of references from identified studies and review articles was also performed to identify additional relevant studies.

### Data abstraction

Two investigators used a common data abstraction tool, but independently abstracted all data. If a disagreement arose it was resolved by a third investigator. The following study-level information was obtained: author identification, year of publication (1990–2000, 2001–2007 or 2008–2013), whether patients were VKA-naïve (<30% of the population receiving a VKA prior to entering the study) or experienced (>70% of the population receiving a VKA prior to entering the study), geographic location of the study (Europe/United Kingdom (UK), Asia, North America, multinational or other), duration of VKA treatment , specific VKA used, interpolation method, whether patients were utilizing VKA self-management to monitor INR control and the study setting (anticoagulation clinic, RCT, or community/standard practice). The setting was designated using the following definitions: an anticoagulation clinic, if the study took place in an anticoagulation clinic or if the stated role of the study clinicians in patient care was limited to managing anticoagulation; a randomized trial if random allocation was employed to assign subjects to receive warfarin or another non-warfarin therapy; and all others were classified as community practice.

Measures of INR control and outcomes were abstracted from each study including TTR, time spent above range, time spent below range, PINRR, proportion of INR measurements above and below therapeutic range and clinical outcomes of major hemorrhage and thromboembolic events. Major hemorrhages included intracranial hemorrhage (ICH) and extracranial bleeding (bleeding requiring hospitalization, blood transfusion or surgical treatment or occurring at a critical anatomical location). All thromboembolic events were abstracted including ischemic stroke, systemic emboli, venous thromboembolism and myocardial infarction. INR measurements at or near each thromboembolic and hemorrhagic event were abstracted when reported.

### Statistical analysis

TTR and PINRR for each study group, as well as the time/proportions below and above range for these measures were expressed as an incidence density using a person-time approach. The numerator was calculated as the proportion of time that the group spent within, below or above the INR range or proportion of INR measurements in, below, or above range multiplied by person-years of follow-up. The denominator was the total person-years of observation for each study group (or the mean/median observation time multiplied by the number of patients in each study group, if person-years of follow-up for the VKA arm(s) in a study was not reported). Ninety-five percent confidence intervals (CIs) were calculated for each incidence density using the Wilson score method without continuity correction. For the purposes of these analyses, all studies were pooled using a random-effects model.

In order to determine how study–level factors influenced TTR or PINRR, both subgroup and meta-regression analyses were conducted. Meta-regression analysis allows evaluating the effect of any given influencing factor independent of the effect of other aspects. A multiple linear mixed method model using both random- and fixed-effects was utilized for meta-regression, which was weighted by the inverse of the variance of TTR or PINRR. Fixed-effects were assumed for study-level factors, including the following covariates: prior experience with VKAs (naïve, experienced, mixed/not reported), study design (community, anticoagulation clinic, RCT), study year (from 1990–2000 and 2001–7, 2008–2013), use of self-management or not, interpolation method (linear or other), geographic region (North America [United States and Canada], Europe/UK, Asia, multinational, other) and duration of VKA treatment (<1 year, ≥1 year). No hierarchy was used in the model for these covariates.

The weighted proportion of thromboembolic and hemorrhagic events occurring outside of the INR range for each study group was also calculated. For this analysis, the numerator was the number of thromboembolic events occurring below an INR of 2.0 or the number of hemorrhagic events above an INR of 3.0. The denominators were the total number of thrombotic or hemorrhagic events for each study group. Ninety-five percent CIs were calculated for each proportion using the Wilson score method without continuity correction. Again, all studies were pooled using a random-effects model. Since not all studies measured INR values at the exact time of adverse outcomes, we conducted two separate analyses, one pooling studies regardless of the elapsed time between the INR measurement and the adverse event; and a second (sensitivity analysis) including only those studies specifically stating an INR was measured within 48 hours of the event.

For all meta-analyses, statistical heterogeneity was determined using the I^2^ statistic (with an I^2^ > 50% signifying an important degree of statistical heterogeneity) and publication bias was assessed using the Egger’s weighted regression statistic (with a p < 0.05 suggesting a higher likelihood of publication bias) and through review of funnel plots (scatterplots of effect size against standard error, where the each dot represents a study effect estimate and the vertical line represents the pooled effect). Statistical analyses were performed using StatsDirect version 2.7.6 (StatsDirect Ltd., Cheshire, England), SAS, version 9.2 (SAS Institute Inc., Cary, NC), and SPSS 15.0 for Windows (SPSS, Inc, Chicago, IL).

## Results

**Figure 1:**
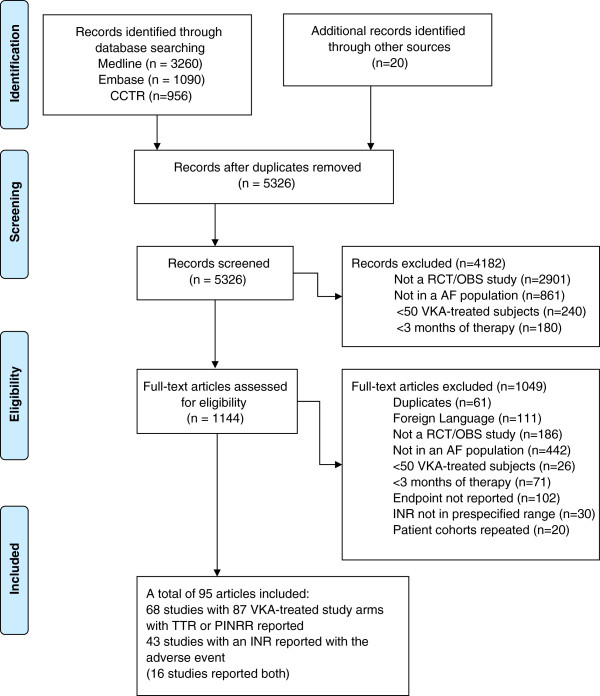
**Flow diagram showing results of the literature search.** CCTR = Cochrane Central Register of Controlled Trials; RCT = randomized controlled trial; OBS = observational; AF = atrial fibrillation; VKA = vitamin K antagonist; INR = international normalized ratio; TTR = time in therapeutic range; PINRR = proportion of INR measurements in range.

We identified 5,326 citations, of which 5,231 were excluded (Figure 1). Ninety-five articles met our inclusion and exclusion criteria [11-103]. Sixty-eight studies reported measures of INR control representing 87 VKA study arms, and 43 studies reported an INR at or near the time of the adverse event (16 studies reported both and were included in both analyses).

Of the 87 VKA study groups that reported a measure of INR control, 17 reported data on patients with no prior VKA exposure whereas 47 enrolled patients who were VKA-experienced and 23 did not report prior VKA exposure data or had mixed enrollment. Twenty-eight VKA groups were from RCTs, 27 were conducted in an anticoagulation clinic setting, and 32 were in a community practice setting (Table 1). Thirty-nine VKA study groups were recruited in Europe/UK, 5 from Asia, 13 were multinational, 1 from Israel and 29 from North America. The mean age ranged from 62 to 92 years (median, 72 years), the number of VKA-treated patients ranged from 55 to 11,770 (median, 249), and the person-years of VKA therapy ranged from 17 to 30,188 (median, 399). Of the 43 identified studies that reported an INR with an adverse event, 16 were RCTs, 14 were conducted in an anticoagulation clinic, and 13 were from community practice with the number of VKA-treated patients ranged from 55 to 9,217 (median, 288) (Table 1).

**Table 1:** Demographics of included studies

**Study, Year**	**Study design/setting**	**VKA-treated N**	**Mean age**	**VKA experience, %**	**Geographic region**	**Interpolation method**	**Self -management**	**Duration of therapy, months (Patient-years)**	**VKA studied**
Abdelhafiz 2004 [11]	PD/AC clinic	402	72	0%	Europe	Linear	N	19 (637)	W
Abdelhafiz (<75) 2008 [12]	PD/AC clinic	203	64	0%	Europe	Linear	N	19 (321)	W
Abdelhafiz (≥75) 2008 [12]		199	80					19 (315)	
Akdeniz 2005 [13]	PD/Community	208	66	NR	Turkey	-	-	-	W
Albers 2005 [14]	RCT	1962	72	85%	N. America	Linear	N	20 (3270)	W
Anderson 2004 [15]	RD/AC clinic	87	NR	100%	N. America	NR	N	12 (87)	W
Ansell (Italy) 2007 [16]	RD/AC clinic	177	72	100%	Multinational	Linear	N	12 (177)	W
Ansell (Spain) 2007 [16]	RD/AC clinic	218	72					12 (218)	A
Ansell (US) 2007 [16]	RD/Community	686	75					12 (686)	W
Ansell (Canada) 2007 [16]	RD/Community	152	74					12 (152)	W
Ansell (France) 2007 [16]	RD/Community	278	73					12 (278)	F
Aronow 1999 [17]	PD/Community	125	83	NR	N. America	NR	N	34 (354)	W
Boulanger 2006 [18]	RD/Community	6454	68	65%	N. America	Linear	N	12 (6454)	W
Burton (<75) 2006 [19]	RD/Community	260	NR	Mixed	Europe	Linear	N	25 (539)	W
Burton (>75) 2006 [19]		341						15 (414)	
Cafolla (Manual) 2011 [20]	RD/Community	576	NR	100%	Europe	Linear	N	8 (384)	A,W
Cafolla 2012 [21]	PD/AC clinic	57	85	100%	Europe	Linear	N	18 (86)	W
Cheung 2005 [22]	RD/Community	555	70	100%	Asia	-	N	19 (893)	W
Chung 2011 [23]	RCT	75	65	55%	Asia	Linear	N	3 (19)	W
Connolly 1991 [24]	RCT	187	68	0%	N. America	Linear	N	15 (234)	W
Connolly 2006 [25]	RCT	3371	70	78%	N. America	Other	N	15 (4214)	NR
Copland 2001 [26]	RD/AC Clinic	328	70	100%	UK	-	N	21 (458)	W
Currie (stable) 2005 [27]	RD/Community	787	74	100%	Europe	Linear	N	35 (2282)	W
Currie (unstable) 2005 [27]		726	78					35 (2105)	
Dentali 2012 [28]	PD/AC Clinic	221	75	100	Europe	-	N	3 (663)	W
DiMarco 2005 [29]	RCT	NR	70	NR	N. America	-	N	42 (−)	W
Dimberg (control SP) 2012 [30]	RD/Community	84	69	100%	Europe	Linear	N	12(84)	W
Dimberg (control ACC) 2012 [30]	RD/AC clinic	271	72					12(271)	
EAFT 1995 [31]	RCT	214	71	0%	Europe	-	N	24 (377)	W,A,F
Ellis 2009 [32]	RCT	66	68	97%	N. America	Linear	N	3 (17)	T
Evans 2000 [33]	PD/AC clinic	288	76	0%	Europe	NR	N	24 (576)	W
Evans 2001 [34]	PD/AC clinic	214	78	NR	Europe	NR	N	24 (448)	W
Ezekowitz 2007 [35]	RCT	70	69	100%	Multinational	NR	N	3 (18)	W
Fang 2004 [36]	RD/AC Clinic	170	78	100	N. America	-	N	-	W
Gallagher 2011 [37]	RD/Community	18113	73	72%	Europe	Linear	N	20 (30188)	W
Garcia (naïve) 2013 [38]	RCT	3888	70	0%	Multinational	Linear	N	22 (7128)	W
Garcia (exp) 2013 [38]		5193	70	100%				22 (9521)	
Gladstone 2009 [39]	RD/Community	423	78	100	N. America	-	N	-	W
Go 2003 [40]	RD/AC clinic	7445	71	Mixed	N. America	Linear	N	21 (12958)	W
Gullov 1998 [41]	RCT	170	73	0%	Europe	Linear	N	25 (355)	W
Gullov 1999 [42]	RCT	170	73	0	Europe	Linear	N	25 (355)	W
Gurwitz 1997 [43]	RD/Community	117	83	32%	N. America	Linear	N	12 (117)	W
Hannon 2011 [44]	PD/Community	43	77	100	Europe	-	N	-	W
Hart 2011 [45]	RCT	523	NR	0	N. America	-	N	13 (575)	W
Heidinger 2000 [46]	RD/Community	753	62	100%	Europe	NR	Y	12 (769)	NR
Hellemons 1999 [47]	RCT	131	70	0	Europe	-	N	32 (354)	P,A
Ho (hypertension) 2011 [48]	RD/Community	278	70	NR	Multinational	Linear	N	48 (1112)	W
Ho (no HTN) 2011 [48]		198	69					48 (792)	
Holmes 2009 [49]	RCT	244	73	100%	Multinational	NR	N	18 (366)	W
Hori 2012 [50]	RCT	250	71	90%	Asia	Linear	N	30 (625)	W
Hylek 2003 [51]	RD/Community	188	76	100	N. America	-	N	-	W
Hylek 2007 [52]	PD/AC clinic	472	77	0%	N. America	Linear	N	12 (360)	W
Jacobs 2009 [53]	RD/Community	90	NR	85%	N. America	Linear	N	12 (90)	W
Jones 2005 [54]	RD/Community	2223	72	100%	Europe	Linear	N	31 (5743)	W
Kalra 2000 [55]	PD/AC clinic	167	77	0%	Europe	Linear	N	24 (296)	W
Kim 2009 [56]	RD/AC clinic	129	64	100%	Asia	Other	N	24 (258)	W
Kim 2010 [57]	PD/AC clinic	499	73	100%	N. America	Linear	N	5 (208)	W
Kulo (warfarin) 2009 [58]	PD/Community	60	66	100%	Europe	NR	N	12 (60)	W
Kulo (acenocoumarol) 2009 [58]		57	68					12 (57)	A
Lee 2012 [59]	RD/Community	200	67	NR	Asia	Linear	N	42 (700)	W
Malik 2000 [60]	RD/AC clinic	247	68	100%	N. America	Other	N	13 (268)	W
Mant 2007 [61]	RCT	488	82	40%	Europe	Linear	N	32 (1301)	W
Matchar (control) 2002 [62]	RCT	317	76	NR	N. America	Linear	N	9 (238)	W
Matchar (prior to ACC) 2002 [62]	PD/AC clinic	363	75					9 (272)	
McBride 2007 [63]	PD/Community	361	71	90%	Europe	Linear	N	9 (271)	W
McCormick 2001 [64]	RD/Community	174	87	100%	N. America	Linear	N	12 (174)	W
Melamed 2011 [65]	RD/Community	906	72	100%	Israel	Linear	N	10 (769)	W
Menzin 2005 [66]	RD/AC clinic	600	72	63%	N. America	Linear	N	11 (525)	W
Morocutti 1997 [67]	RCT	454	72	NR	Europe	-	N	12	W
Neree 2006 [68]	RD/Community	395	74	100%	Europe	Linear	N	4 (132)	P,A,W
Nichol (ACC) 2008 [69]	RD/AC clinic	351	NR	100%	N. America	Linear	N	31 (920)	W
Nichol (UC) 2008 [69]	RD/Community	756						18 (1165)	
Njaastad 2006 [70]	RD/AC clinic	421	NR	0%	Europe	Linear	N	14 (475)	W
Nozawa 2001 [71]	PD/Community	156	68	100	Asia	-	N	22 (286)	W
Ogawa 2011 [72]	RCT	75	72	0	Asia	Linear	N	3 (210)	W
Okumura (<70) 2011 [73]	PD/Community	208	NR	100%	Asia	Linear	N	23 (399)	W
Olsson 2003 [74]	RCT	1703	70	73%	Multinational	NR	N	16 (2271)	W
Ono 2005 [75]	PD/Community	63	76	0	Asia	Linear	N	28 (145)	W
Patel 2011 [76]	RCT	7133	73	63%	Multinational	Linear	N	20 (11888)	W
Pengo 1998 [77]	RCT	153	74	0%	Europe	Linear	N	14 (179)	W
Pengo 2001 [78]	PD/AC Clinic	433	68	0	Europe	Linear	N	17 (615)	W,A
Pengo 2010 [79]	RCT	132	79	0%	Multinational	Linear	N	64 (704)	W
Perez-Gomez 2004 [80]	RCT	237	70	0%	Europe	NR	N	33 (556)	A
Poli 2007 [81]	PD/AC clinic	290	82	100%	Europe	Linear	N	34 (814)	NR
Poli 2009 [82]	PD/AC Clinic	783	75	0%	Europe	Linear	N	39 (2567)	W
Poli 2009 [83]	PD/AC clinic	578	75	100%	Europe	Linear	N	38 (1854)	NR
Poli 2009 [84]	PD/AC clinic	780	75	100	Europe	Linear	N	36 (2406)	W
Poller (Manual) 2008 [85]	RCT	2967	67	0%	Multinational	Linear	N	17 (4203)	W, A, P
Sadanaga 2010 [86]	PD/Community	259	74	100	Asia	-	N	25 (544)	W
Samsa (ACC) 2000 [87]	RD/AC Clinic	43	69	NR	N. America	Linear	N	9 (32)	W
Samsa (Rochester) 2000 [87]	RD/Community	61						9 (46)	
Samsa (R. Triangle) 2000 [87]	RD/Community	125						9 (94)	
Schwammenthal 2010 [88]	PD/Community	111	76	100	Israel	-	N	-	W
Sconce (vitamin K) 2007 [89]	RCT	35	75	100%	Europe	Linear	N	6 (18)	W
Sconce (placebo) 2007 [89]		35	74						
Shen 2007 [90]	RD/Community	NR	72	55%	N. America	NR	N	40 (24179)	W
Singer 2009 [91]	RD/Community	9217	77	100	N. America	-	N	72 (33497)	W
SPAF III 1996 [92]	RCT	523	71	56%	N. America	NR	N	13 (567)	W
Sullivan (women) 2012 [93]	RCT	1594	71	87%	Multinational	Linear	N	42 (5579)	W
Sullivan (men) 2012 [93]		2466	68	86%				42 (8631)	
Tincani 2009 [94]	PD/AC Clinic	90	92	100%	Europe	Linear	N	12 (90)	W
Van Spall 2012 [95]	RCT	6022	72	47%	Multinational	Linear	N	24 (12044)	W
Voller (self-mgmt) 2005 [96]	RCT	101	65	0%	Europe	NR	Y	8 (68)	NR
Voller (UC) 2005 [96]		101	64				N		
Walker (MHC) 2011 [97]	RD/AC Clinic	22	NR	100%	N. America	Linear	N	12 (22)	W
Walker (no MHC) 2011 [97]		62						12 (62)	
Wieloch (AF) 2011 [98]	RD/Community	11770	73	100%	Europe	Linear	N	12 (11770)	W
Weitz 2010 [99]	RCT	250	66	35%	N. America	Linear	N	3 (63)	W
Wyse 2002 [100]	RCT	NR	70	NR	N. America	-	N	42	W
Yamaguchi 2000 [101]	RCT	55	66	NR	Asia	-	N	22 (101)	W
Yasaka 2001 [102]	PD/Community	88		100	Asia	-	N	22 (161)	W
Yousef 2004 [103]	RD/Community	739	73	100%	Europe	NR	N	24 (1484)	W

VKA = vitamin K antagonist; RCT = randomized controlled trial; RD = retrospective design; PD = prospective design; AC clinic = anticoagulation clinic; AF = atrial fibrillation; NR = not reported; N = North; VKA = Vitamin K Antagonist; W = warfarin; A = acenocoumarol; T = tecarfarin; P = phenprocoumon; F = fenprocoumon.

For the first meta-analysis, 78 out of 87 (90%) study groups reported a TTR and 55 (63%) reported the time spent below and above range; 22 of 87 (25%) study groups reported a PINRR and 21 (24%) reported the percent of INR measures below and above range (Table 2). Thirteen study groups (15%) reported both TTR and PINRR. Overall, patients spent 61% of their TTR (95% CI, 59–62%), 25% (95% CI, 23–27%) below, and 14% (95% CI, 13-15%) above range. Similarly for PINRR, 56% of INR measurements were in the therapeutic range (95% CI, 53–59%), 26% (95% CI, 23–29%) below and 13% (95% CI, 11-17%) above range. Statistical heterogeneity was observed to be high between the groups included in these analyses (I^2^ ≥ 97%). The likelihood of publication bias appeared low for TTR, time above range, PINRR, and proportion of INR measurements below and above range. However, publication bias was deemed more likely for the time below range analysis (Egger’s p = 0.03) (Additional file 1).

**Table 2:** Time in range and percentage of INR values in range and major adverse event rates

**Study, year**	**N**	**TTR, %**			**PINRR, %**			**Event rate, % per patient-year**		
		**In range**	**Below range**	**Above range**	**In range**	**Below range**	**Above range**	**Bleeding**	**ICH**	**Ischemic stroke**
Abdelhafiz 2004 [11]	402	66	19.7	14.3	-	-	-	1.7	-	-
Abdelhafiz (<75) 2008 [12]	203	58	16	26	-	-	-	1.6	0	-
Abdelhafiz (≥75) 2008 [12]	199	58	16	26	-	-	-	1.9	0	-
Albers 2005 [14]	1962	68	20	12	-	-	-	2.8	0.2	1.1
Anderson 2004 [15]	87	-	-	-	60.4	25.2	14.3	-		
Ansell (Italy) 2007 [16]	177	68.9	21	10.1	60	26.3	13.6	-	-	-
Ansell (Spain) 2007 [16]	218	64.4	18.6	17	59	23.3	17.7	-	-	-
Ansell (US) 2007 [16]	686	57	29.1	13.9	50.8	32	17.3	-	-	-
Ansell (Canada) 2007 [16]	152	61	25.9	13.1	58.4	27.6	14	-	-	-
Ansell (France) 2007 [16]	278	58.1	15.4	26.5	51.3	19.7	29	-	-	-
Aronow 1999 [17]	125	-	-	-	68	26	6	-	-	11.3
Boulanger 2006 [18]	6454	48	38	14	-	-	-	2.8	-	-
Burton (<75) 2006 [19]	260	68	17	15	-	-	-	2.8		
Burton (>75) 2006 [19]	341	68	19	13	-	-	-	2.4		
Cafolla (Manual) 2011 [20]	576	60.4	-	-	-	-	-	-	-	-
Cafolla 2012 [21]	57	64.4	-	-	-	-	-	0	-	-
Chung 2011 [23]	75	45.1	43.9	10.9	-	-	-	10.5	0	0
Connolly 1991 [24]	187	43.7	39.6	16.6	-	-	-	2.1	0.4	2.1
Connolly 2006 [25]	3371	63.8	20.8	15.4	-	-	-	2.2	-	1.0
Currie (stable) 2005 [27]	787	74.9	-	-	-	-	-	0.4	-	-
Currie (unstable) 2005 [27]	726	55.7	-	-	-	-	-	1.2	-	-
Dimberg (UC) 2012 [30]	84	64.3	-	-	-	-	-	-	-	-
Dimberg (ACC) 2012 [30]	271	73.6	-	-	-	-	-	-	-	-
Ellis 2009 [32]	66	71.4	-	-	70.7	16.0	13.3	5.8		
Evans 2000 [33]	288	55	26	19	-	-	-	3.1	0.5	5.5
Evans 2001 [34]	214	66	-	-	-	-	-	3.1	0.7	4.2
Ezekowitz 2007 [35]	70	57.2	-	-	-	-	-	0	-	-
Gallagher 2011 [37]	18113	63.1	20.7	16.2	-	-	-	-	-	-
Garcia (naïve) 2013 [38]	3888	61.4	-	-	-	-	-	3.0	0.8	-
Garcia (exp) 2013 [38]	5193	69.1	-	-	-	-	-	3.2	0.8	-
Go 2003 [40]	7445	62.5	26.8	10.7	-	-	-	1.5	0.4	1.1
Gullov 1998 [41]	170	73	18	9	-	-	-	1.1	0.6	0.8
Gurwitz 1997 [43]	117	39.6	44.8	15.6	-	-	-	-	-	-
Heidinger 2000 [46]	753	-	-	-	69.5	23.5	7.0	1.7	-	-
Ho (HTN) 2011 [48]	278	49.7	45.4	4.9	-	-	-	1.7	0.5	2.2
Ho (no HTN) 2011 [48]	198	49.8	44.2	6.0	-	-	-			
Holmes 2009 [49]	244	66	-	-	-	-	-	2.7	-	1.6
Hori 2012 [50]	250	-	-	-	51.8	43.8	4.2	0.8		0.3
Hylek 2007 [52]	472	58	29	11	-	-	-	7.2	2.5	-
Jacobs 2009 [53]	90	49	35	15	-	-	-	5.6		
Jones 2005 [54]	2223	68.9	16.7	15.4	53.4	-	-	-	-	-
Kalra 2000 [55]	167	61	26	13	-	-	-	1.4	0.3	2.0
Kim 2009 [56]	129	-	-	-	60.9	31.2	9.1	0		
Kim 2010 [57]	499	73.4	-	-	-	-	-	-	-	-
Kulo (warfarin) 2009 [58]	60	-	-	-	51.8	42.8	5.4	-	-	-
Kulo (acenocoum) 2009 [58]	57	-	-	-	53.6	35.9	10.5	-	-	-
Lee 2012 [59]	200	48.5	-	-	-	-	-	6.6	-	-
Malik (AF) 2000 [60]	247	58.9	27.4	14.1	-	-	-	-	-	-
Mant 2007 [61]	488	67	19	14	-	-	-	1.9	-	0.8
Matchar (control) 2002 [62]	317	52.3	31.8	15.9	-	-	-	2.1	-	-
Matchar (ACC) 2002 [62]	363	55.6	31.2	13.1	-	-	-	2.2	-	-
McBride 2007 [63]	361	56	16	28	-	-	-	-	-	-
McCormick 2001 [64]	174	51	36	13	-	-	-	-	-	-
Melamed 2011 [65]	906	48.6	32.0	19.3	-	-	-	-	-	-
Menzin 2005 [66]	600	62	25	13	-	-	-	3.6	-	1.0
Neree 2006 [68]	395	53	-	-	52.8	8.7	38.5	4.4	-	-
Nichol (ACC) 2008 [69]	351	68	21	11	-	-	-	2.3	-	-
Nichol (UC) 2008 [69]	756	42	49	9	-	-	-	6.3	-	-
Njaastad 2006 [70]	421	71.5	-	-	-	-	-	0.8	-	-
Okumura (<70) 2011 [73]	208	46	51	2	-	-	-	-	-	-
Olsson 2003 [74]	1703	66	-	-	-	-	-	2.2	-	1.9
Patel 2011 [76]	7133	55	-	-	-	-	-	3.4	0.7	-
Pengo 1998 [77]	153	70	18	12	-	-	-	2.8	0.6	0
Pengo 2010 [79]	132	65	25	9	-	-	-	3.0	-	-
Perez-Gomez 2004 [80]	237	65	19	16	-	-	-	1.8		
Poli 2007 [81]	290	69	15	16	-	-	-	2.1	1.4	-
Poli 2009 [82]	783	71	14	15	-	-	-	1.4	0.7	0.9
Poli 2009 [83]	578	68	16	16	-	-	-	-	-	-
Poller (Manual) 2008 [85]	2967	66.2	-	-	-	-	-	-	-	-
Samsa (ACC) 2000 [87]	43	60.3	25.9	13.8	54.9	26.7	18.3	-	-	-
Samsa (Rochester) 2000 [87]	61	46.9	33.9	19.3	43.6	33.5	22.9	-	-	-
Samsa (R. Triangle) 2000 [87]	125	35.6	52.1	12.2	34.2	51.0	14.8	-	-	-
Sconce (vitamin K) 2007 [89]	35	87	-	-	-	-	-	-	-	-
Sconce (placebo) 2007 [89]	35	78	-	-	-	-	-	-	-	-
Shen 2007 [90]	NR	54.5	36.3	9.2	-	-	-	-	0.5	-
SPAF III 1996 [92]	523	-	-	-	61	25	14			
Sullivan (women) 2012 [93]	1594	60	29	11	-	-	-	-	-	0.8
Sullivan (men) 2012 [93]	2466	63	26	14	-	-	-	-	-	0.5
Tincani 2009 [94]	90	66	19	14	-	-	-	5.5	3.3	2.2
Van Spall 2012 [95]	6022	64	22	13	-	-	-	-	-	-
Voller (self-mgmt) 2005 [96]	101	72.4	13.6	14	67.8	15.2	17.0	2.9	-	-
Voller (UC) 2005 [96]	101	63.6	19.5	16.9	58.5	22.1	19.4	0	-	-
Walker (MHC) 2011 [97]	22	56.8	34.2	9.0	-	-	-	-	-	-
Walker (no MHC) 2011 [97]	62	65.9	30.7	3.4	-	-	-	-	-	-
Wieloch (AF) 2011 [98]	11770	76.5	-	-	-	-	-	2.6	-	-
Weitz 2010 [99]	250	49.7	-	-	-	-	-	1.6	-	-
Yousef 2004 [103]	739	-	-	-	62.8	22.9	14.3	1.9	-	-

AC = Anticoagulation; AF = atrial fibrillation; ICH = Intracranial Hemorrhage; HTN = Hypertension, INR = international normalized ratio; MGMT = Management; N = Number; SP = Standard Practice; TTR = time in the therapeutic range; PINRR = proportion of INR Measurements in range.

Upon meta-regression to determine how study-level factors influenced TTR, community VKA management was associated with less time in range than therapy managed in an anticoagulation clinic or RCT setting, European/UK study patients spent more time in range than North American study patients, and the VKA-naïve (and mixed/not reported) spent less time in range than VKA-experienced patients (Table 3). For PINRR, no study-level factor was found to be significantly different than the referent upon meta-regression (p > 0.05) (Table 4).

**Table 3:** Results of traditional meta-analysis and meta-regression analyses of time in the therapeutic range

		**Time spent in therapeutic range**		
**Study-level factors**	**No. (%)**	**Unadjusted pooled mean upon subgroup analysis, % (95% CI)**	**Adjusted difference, % (95% CI)**	***p-v*** **alue**
All study groups	78 (100)	61 (59 to 62)	NA	NA
Study setting				
AC Clinic	25 (32.1)	64 (62 to 66)	7.2 (3.2 to 11.2)	<0.001
RCT	26 (33.3)	63 (61 to 65)	9.1 (4.3 to 13.9)	<0.001
Community	27 (34.6)	55 (52 to 59)	Referent	
Study year				
1990-2000	10 (12.8)	53 (46 to 61)	−4.3 (−10.2 to 1.6)	0.16
2001-2007	34 (43.6)	64 (62 to 65)	2.2 (−1.7 to 6.1)	0.28
2008-2013	34 (43.6)	60 (57 to 63)	Referent	
Interpolation method				
NR/Other	11 (14.1)	63 (60 to 65)	−2.1 (−6.8 to 2.6)	0.38
Linear	67 (85.9)	60 (59 to 62)	Referent	
Self-management				
No	77 (98.7)	60 (58 to 62)	−6.8 (−23.7 to 10.1)	0.43
Yes	1 (1.3)	72 (68 to 77)	Referent	
Geographic region				
Europe/UK	35 (44.9)	67 (64 to 69)	9.7 (6.0 to 13.4)	<0.001
Asia	3 (3.8)	47 (44 to 49)	−5.5 (−14.7 to 3.7)	0.24
Other	1 (1.3)	49 (46 to 52)	−3.0 (−16.1 to 10.1)	0.65
Multinational	13 (16.7)	61 (57 to 65)	1.8 (−4.1 to 7.7)	0.55
North America	26 (33.3)	55 (50 to 60)	Referent	
VKA experience				
NR/Mixed	21 (26.9)	54 (49 to 60)	−4.6 (−8.3 to −0.9)	0.02
No	17 (21.8)	63 (60 to 66)	−5.3 (−9.6 to −1.0)	0.02
Yes	40 (51.3)	63 (60 to 66)	Referent	
Duration of VKA treatment				
≥1 year	59 (75.6)	62 (60 to 63)	2.7 (−1.7 to 7.1)	0.23
<1 year	19 (24.4)	58 (52 to 65)	Referent	

AC = anticoagulation; CI = confidence interval; NA = not applicable; No. = number of study arms; NR = Not Reported; RCT = randomized controlled trial; UK = United Kingdom, VKA = Vitamin K Antagonists.

**Table 4:** Results of traditional meta-analysis and meta-regression analyses of proportion of INR measurements in range

		**Proportion of INR measurements in range**		
**Study-level factors**	**No. (%)**	**Unadjusted pooled mean upon subgroup analysis, % (95% CI)**	**Adjusted difference, % (95% CI)**	***p-v*** **alue**
All study groups	24 (100)	56 (53 to 59)	NA	NA
Study setting				
AC Clinic	5 (20.8)	60 (58 to 62)	6.9 (−0.02 to 15.2)	0.13
RCT	5 (20.8)	61 (54 to 68)	5.6 (−0.06 to 17.0)	0.36
Community	14 (58.3)	54 (50 to 57)	Referent	
Study year				
1990-2000	6 (25.0)	54 (46 to 64)	3.3 (−25.3 to 31.9)	0.82
2001-2007	13 (54.2)	56 (54 to 59)	−2.8 (−14.8 to 9.2)	0.65
2008-2013	5 (20.8)	56 (51 to 62)	Referent	
Self-management				
No	22 (91.7)	55 (43 to 57)	−9.3 (−32.4 to 13.8)	0.45
Yes	2 (8.3)	69 (67 to 72)	Referent	
Geographic region				
Europe/UK	13 (54.2)	57 (54 to 60)	−1.7 (−10.7 to 7.3)	0.71
Asia	2 (8.3)	56 (48 to 66)	−8.8 (−26.4 to 8.8)	0.32
Other	0 (0)	NA	NA	NA
Multinational	0 (0)	NA	NA	NA
North America	9 (37.5)	54 (48 to 62)	Referent	
VKA experience				
NR/Mixed	5 (20.8)	51 (41 to 63)	−5.7 (−34.7 to 23.3)	0.83
No	3 (12.5)	63 (55 to 73)	7.9 (−15.2 to 31.0)	0.65
Yes	17 (70.8)	56 (54 to 59)	Referent	
Duration of VKA treatment				
≥1 year	17 (70.8)	57 (54 to 60)	9.1 (−0.5 to 18.7)	0.09
<1 year	7 (29.2)	53 (44 to 65)	Referent	

AC = anticoagulation; CI = confidence interval; NA = not applicable; No. = number of study arms; NR = not reported; RCT = randomized controlled trial; UK = United Kingdom; VKA = Vitamin K Antagonists.

In the second meta-analysis, 30 of 43 studies (70%) reported an INR measure with thromboembolic events, 21 (49%) reported an INR with ischemic stroke, 32 (74%) reported INRs with major hemorrhage, and 16 (37%) reported an INR with ICH. INR data was incomplete in 58% of the studies that reported adverse events; not all events were reported with an INR measure. Thirty percent of the studies used previously reported INRs, allowing INR measures from 3 days up to 12 days prior to be considered as temporally related to the adverse event.

**Figure 2:**
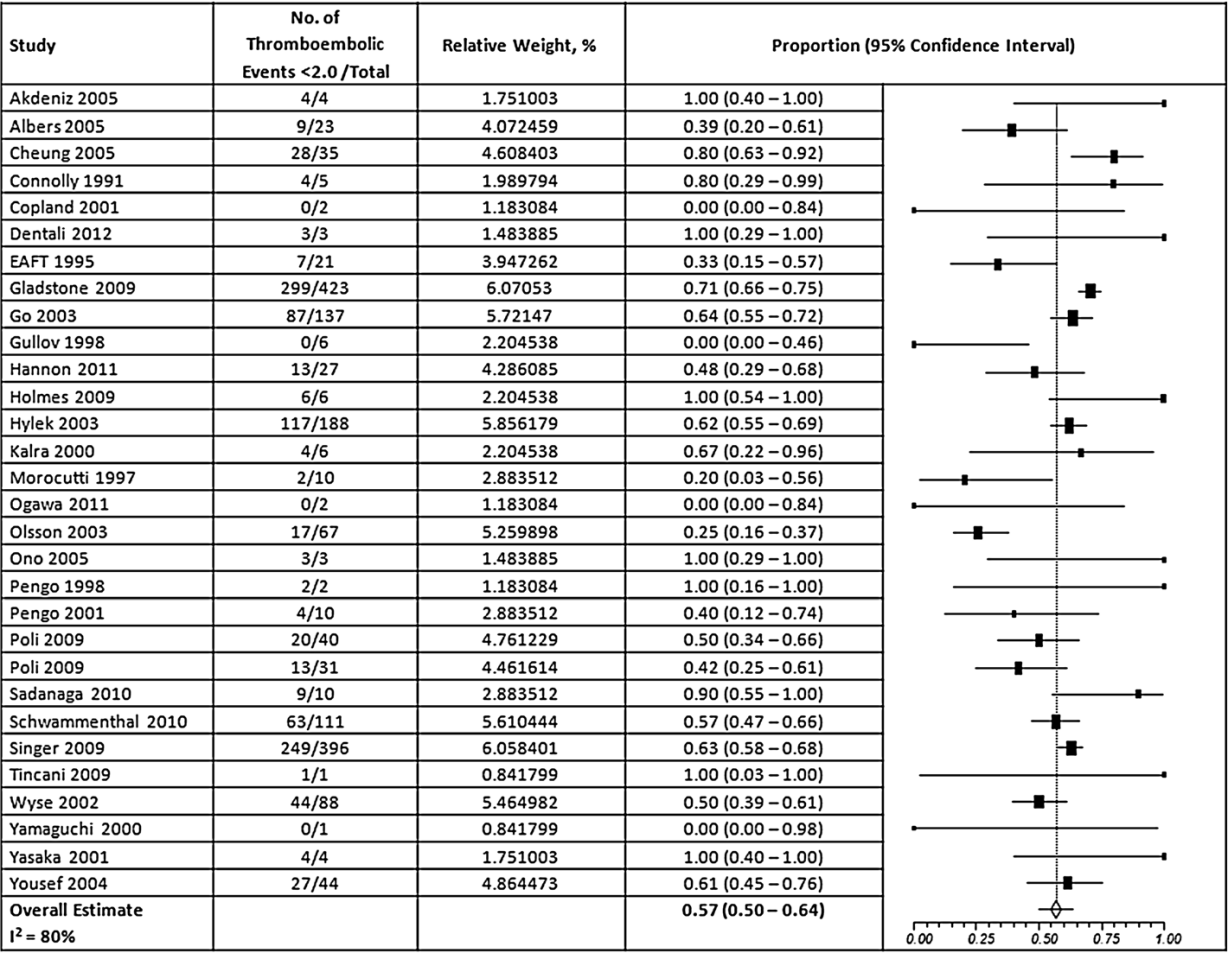
**Results of random-effects meta-analysis of the proportion of thromboembolic events that occurred when INRs were below 2.0.** The squares represent individual studies, and the size of the square represents the weight given to each study in the meta-analysis. Error bars represent 95% confidence intervals. The diamond represents the combined results. List of studies shows name of first author and year of publication. CI = Confidence Interval.

**Figure 3:**
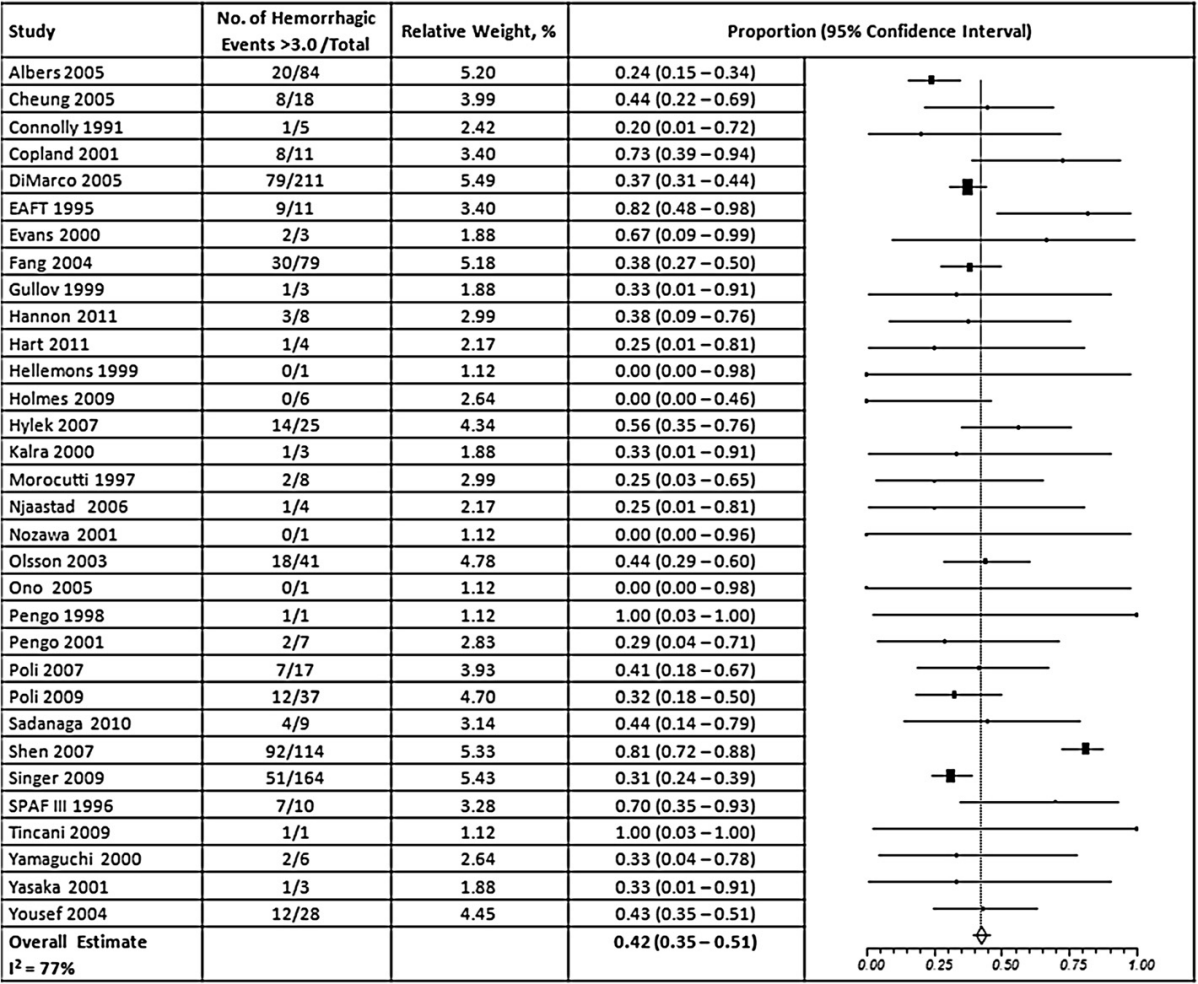
**Results of random-effects meta-analysis of the proportion of major hemorrhagic events that occurred when INRs were above 3.0.** The squares represent individual studies, and the size of the square represents the weight given to each study in the meta-analysis. Error bars represent 95% confidence intervals. The diamond represents the combined results. List of studies shows name of first author and year of publication. CI = Confidence Interval.

**Figure 4:**
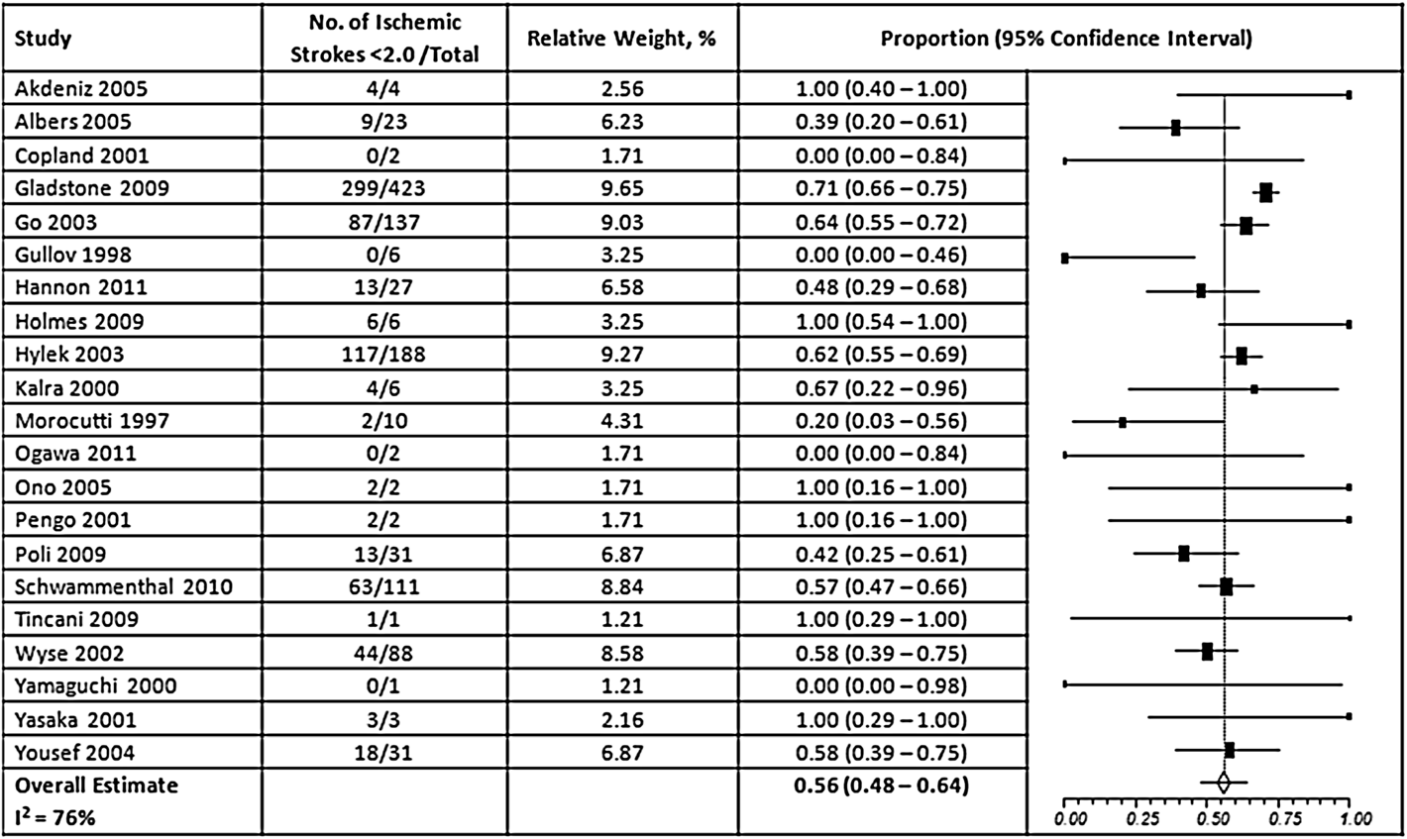
**Results of random-effects meta-analysis of the proportion of ischemic strokes that occurred when INRs were below 2.0.** The squares represent individual studies, and the size of the square represents the weight given to each study in the meta-analysis. Error bars represent 95% confidence intervals. The diamond represents the combined results. List of studies shows name of first author and year of publication. CI = Confidence Interval.

**Figure 5:**
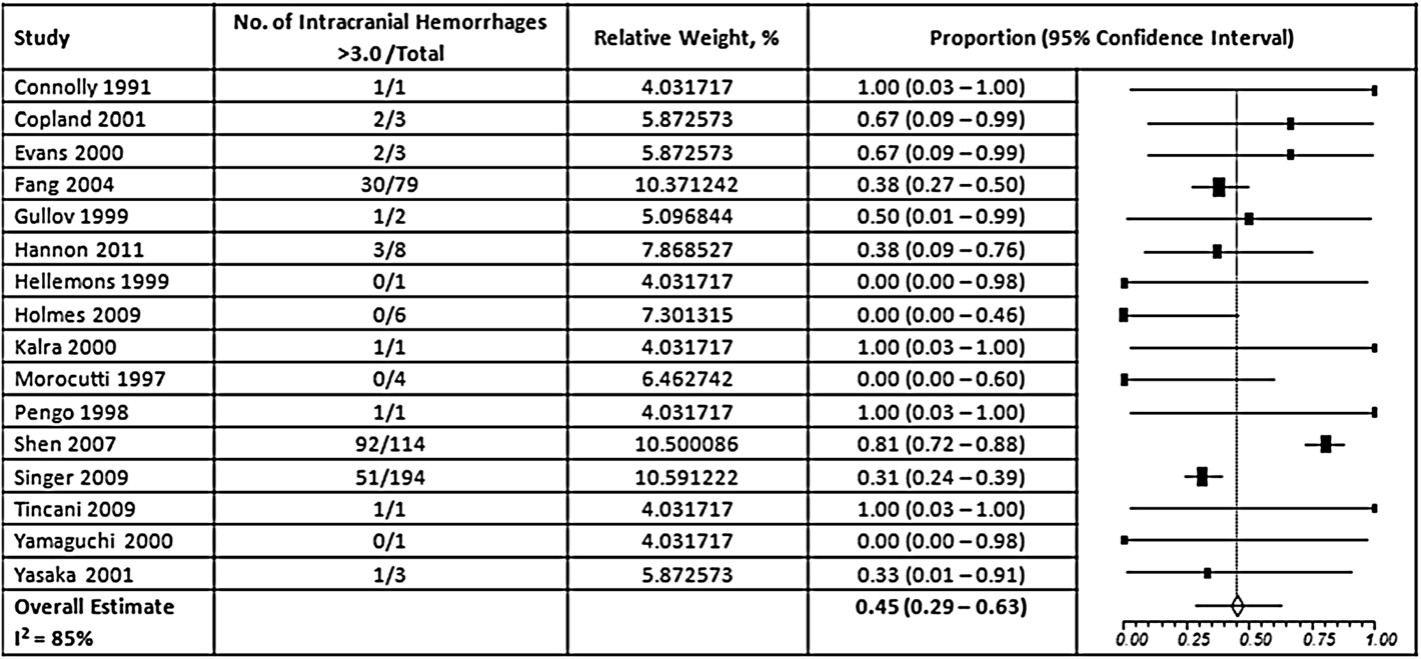
**Results of Random-Effects Meta-Analysis of the Proportion of Intracranial Hemorrhages That Occurred When INRs Were Above 3.0.** The squares represent individual studies, and the size of the square represents the weight given to each study in the meta-analysis. Error bars represent 95% confidence intervals. The diamond represents the combined results. List of studies shows name of first author and year of publication. CI = Confidence Interval; ICH = Intracranial hemorrhage.

Overall, 57% of the thromboembolic events occurred at an INR <2.0 (95% CI, 50-64%) and 42% of hemorrhagic events occurred at an INR >3.0 (95% CI, 35 – 51%) (Figures 2 and 3). A high degree of heterogeneity was present for studies that reported thromboembolic and hemorrhagic events (I^2^ = 80% and 77% respectively); however, the presence of publication bias was deemed low (Egger’s p = 0.31 and p = 0.69, respectively) (Additional file 1). When ischemic stroke and ICH were evaluated separately from other events, we found that 56% of ischemic strokes (95% CI, 48 – 64%) and 45% of ICHs (95% CI, 29-63%) occurred at INRs <2.0 and >3.0, respectively, but a high degree of heterogeneity was still present (I^2^ = 76% and 85%, respectively) (Figures 4 and 5). Furthermore, when studies that allowed previously reported INRs (more than 48 hours prior to an event) were excluded, we found a higher proportion of events that occurred outside the therapeutic range for 59% of thromboembolic events (95% CI, 51 – 66%) and 47% of hemorrhagic events (95% CI, 37 – 58%).

## Discussion

In a pooled analysis of AF studies performed worldwide between 1990 and June 2013, we found patients spent only 61% of their TTR and only 56% of their measured INRs were in range. Moreover, when patients were out of range, they were more likely to be below range and at an increased risk of thrombosis, as compared to being above range with an increased risk of bleeding. While, thromboembolic and hemorrhagic events did occur in patients with a therapeutic INR; we demonstrated more than half of all thromboembolic events occurred when the INR was less than 2.0 and more than 40% of all hemorrhagic events happened at an INR > 3.0. As such, the efficacy and safety of VKAs appear strongly tethered to the quality of INR control achieved.

Our meta-regression analyses showed three important findings. First, patients having their VKAs managed in the community setting spent significantly less TTR when compared to patients managed in an anticoagulation clinic or in a RCT setting. This data supports the growing body of literature demonstrating that patients whose care is managed in an anticoagulation clinic have better outcomes (improved TTR, lower rates of major bleeding and thrombosis, and decreased health care costs) than those managed in community practice [104-106]. The improved INR control in RCTs and anticoagulation clinics may be the result of increasing the frequency of monitoring, providing more organized care and focusing more on improving poor VKA-drug persistence (as low as 68% at 6-months) [107,108] than typically done in community settings. Unfortunately, not all patients receiving a VKA have access to anticoagulation clinics [109]. In fact, it is estimated that only about one-third of patients receiving a VKA in the US have access to an anticoagulation clinic due to time, distance, economic or other access-to-care issues. Second, patients who were VKA-naive spent significantly less time in the therapeutic range than VKA-experienced patients. This is likely a result of poorer INR control early on in treatment, as clinicians attempt to control the INR within the narrow therapeutic window without any prior knowledge of the patient’s-specific dosing requirements. It may also suggest that patients learn to manage their VKA better over time. This observation may warrant researchers to stratify analyses according to whether patients are newly initiated to warfarin or experienced warfarin patients. Finally, people in North America spent significantly less time in the therapeutic range than those in Europe or the UK. This may be a result of North America’s near exclusive use of warfarin, which has been shown in previous analyses to result in as much as a 9% lower TTR compared to other VKAs [6], as well as, North America’s less wide-spread use of proven strategies to increase TTR such as and anticoagulation clinics as noted above [6,8,9,109] and patient self-monitoring [6].

Our systematic review and meta-analysis supports and extends the knowledge of VKA use in contemporary practice. Our TTR or PINRR results are in general agreement with the systematic reviews by van Walraven et al. [6] and Cios et al. [9], but these analyses included all therapeutic indications for VKAs, which lowers their applicability for an AF-specific population. The meta-analyses by Baker et al. [8] and Wan et al. [7] focused on AF only populations, but each had some limitations. The Baker meta-analysis was limited to a United States population, and therefore, contained only 8 studies. Despite this, they did find that care within an anticoagulation clinic yielded a higher TTR than usual care in the community, a finding we extend into the worldwide AF population as well. The Wan meta-analysis was published in 2008, evaluated a worldwide AF population and found similar TTR and PINRR results to our own. However, they only included 47 VKA groups from 38 published studies. They found that TTR was significantly correlated with PINRR in retrospective studies and TTR was significantly negatively correlated to both major hemorrhage and thromboembolic events. Given the intense focus on thromboembolism in contemporary practice, our literature search was able to almost double the number of articles included in the Wan et al. by updating the search to 2013. Over the 5 years since the publication of Wan et al.’s meta-analysis, we found the TTR and PINRR to be only slightly increased (overall TTR increased from 57% to 61% and PINRR from 51 to 56%) [7]. This suggests that we still have a long way to go to enhance the quality of INR control. Our findings that the majority of thromboembolic events happen in AF patients when the INR is less than 2.0 and more than 40% of hemorrhages occur when an INR is greater than 3.0 confirms the findings from the systematic review of Oake et al. [10]. Their systematic review did not limit the studies to an AF population and was published in 2009, so our findings through June 2013 in the target AF population extend knowledge in this area as well.

There are several limitations of our meta-analysis worth discussion. First, the potential for publication bias and possibility of missed eligible articles could exist. However, we consider this risk to be minimized due to our systematic search strategy and manual backwards citation tracking. In addition, having such a large cadre of studies, as we do in our meta-analysis, minimizes the impact that a missed individual study might have on our pooled result. Another limitation of our analysis includes the fact that very few identified studies evaluated PINRR. As a result, our meta-regression analysis was likely underpowered; although similar trends could be seen to TTR. Future evaluation of PINRR should be conducted when there is a sufficient literature base to make firmer conclusions. Another limitation of our analysis is the possibility of a language bias; as we only included English language studies which may not represent all of the published evidence. Finally, there was a high degree of statistical heterogeneity observed in our analyses, suggesting that the included studies varied clinically and/or methodologically. However, this was one of our rationales for conducting meta-regression. In our meta-regression analyses, we found some potential explanations for the identified heterogeneity; although there may still be additional factors that we could not think to evaluate or for which there is insufficient data to allow evaluation.

## Conclusions

Patients on VKAs for thromboembolism prevention in AF are frequently outside the normal INR range and tend to be under-anticoagulated rather than over-anticoagulated. While, thromboembolic and hemorrhagic events do occur patients with a therapeutic INR; patients with an INR < 2.0 make up many of the cases of thromboembolism, while those with an INR > 3.0 make up many of the cases of major hemorrhage. Managing anticoagulation outside of a clinical trial or anticoagulation clinic is associated with poorer INR control as is initiation of therapy in patients who are VKA-naïve. Patients in Europe/UK have better INR control than those in North America.

## Abbreviations

AF: Atrial fibrillation; VKA: Vitamin K antagonist; TTR: Time in therapeutic range; PINRR: Proportion of INR measurements in range; INR: International normalized ratio; CI: Confidence interval; UK: United Kingdom; RCT: Randomized controlled trial; ICH: Intracranial hemorrhage.

## Competing interests

Dr. Coleman has received honoraria for participation on advisory boards and Speaker’s bureaus and has received research funding from Janssen Scientific Affairs. Drs Raut and Schein are paid employees of Janssen Pharmaceuticals. Drs. Mearns, White and Kohn and Mr./Ms. Hawthorne, Song and Meng have no conflicts to report.

## Authors’ contributions

Study concept and design: ESM, MKR, JRS, CIC. Acquisition of data: ESM, CGK, JH, JSS, JM. Analysis and interpretation of data: ESM, CMW, CGK, JH, JSS, JM, MKR, JRS, CIC. Drafting of the manuscript: ESM, CMW, CGK, CIC. Critical revision of the manuscript for important intellectual content: ESM, CMW, MKR, JRS, CIC. Administrative, technical, or material support: ESM, MKR, JRS, CIC. Study supervision: ESM, CMW, CIC. ESM and CIC had full access to all the data in the study and take responsibility for the integrity of the data and the accuracy of the data analysis. All authors read and approved the final manuscript.

## Supplementary Material

Additional file 1 Medline search strategy and forest plots for time and percent of INRs in, below and above range. Click here for file
