# Supplementary material for: Quality of vitamin K antagonist control and outcomes in atrial fibrillation patients: a meta-analysis and meta-regression
Source: Thromb J. 2014 Jun 24;12:14. doi: 10.1186/1477-9560-12-14 (PMC4094926; doi:10.1186/1477-9560-12-14)
Supplement: Additional file 1 — Medline search strategy and forest plots for time and percent of INRs in, below and above range. [file 1477-9560-12-14-S1.docx]

**DATA SUPPLEMENT**

**eAppendix. Medline Search Strategy**

(("atrial fibrillation"[MeSH Terms] OR ("atrial"[All Fields] AND "fibrillation"[All Fields]) OR "atrial fibrillation"[All Fields]) OR ("venous thromboembolism"[MeSH Terms] OR ("venous"[All Fields] AND "thromboembolism"[All Fields]) OR "venous thromboembolism"[All Fields]) OR VTE[All Fields]) AND (("international normalised ratio"[All Fields] OR "international normalized ratio"[MeSH Terms] OR ("international"[All Fields] AND "normalized"[All Fields] AND "ratio"[All Fields]) OR "international normalized ratio"[All Fields]) OR VKA[All Fields] OR (vitamin k antagonism[All Fields] OR vitamin k antagonist[All Fields] OR vitamin k antagonisten[All Fields] OR vitamin k antagonists[All Fields]) OR ("warfarin"[MeSH Terms] OR "warfarin"[All Fields]) OR ("acenocoumarol"[MeSH Terms] OR "acenocoumarol"[All Fields]) OR ("dicumarol"[MeSH Terms] OR "dicumarol"[All Fields]) OR ("ethyl biscoumacetate"[MeSH Terms] OR ("ethyl"[All Fields] AND "biscoumacetate"[All Fields]) OR "ethyl biscoumacetate"[All Fields]) OR ("phenprocoumon"[MeSH Terms] OR "phenprocoumon"[All Fields]) OR ("4-hydroxycoumarins"[MeSH Terms] OR "4-hydroxycoumarins"[All Fields] OR "4 hydroxycoumarins"[All Fields])) AND ((Clinical Trial[ptyp] OR Clinical Trial, Phase I[ptyp] OR Clinical Trial, Phase II[ptyp] OR Clinical Trial, Phase III[ptyp] OR Comparative Study[ptyp] OR Controlled Clinical Trial[ptyp] OR Evaluation Studies[ptyp] OR Meta-Analysis[ptyp] OR Multicenter Study[ptyp] OR Randomized Controlled Trial[ptyp] OR Research Support, N I H, Extramural[ptyp] OR Research Support, Non U S Gov't[ptyp] OR Research Support, U S Gov't, Non P H S[ptyp] OR Research Support, U S Gov't, P H S[ptyp] OR Research Support, U.S. Government[ptyp] OR Validation Studies[ptyp]) AND ("1990/01/01"[PDAT] : "3000/12/31"[PDAT]) AND "humans"[MeSH Terms] AND English[lang])

Egger bias P=0.23

*-1.1*

*-0.9*

*-0.7*

*-0.5*

*-0.3*

*-0.1*

*0.12*

*0.09*

*0.06*

*0.03*

*0.00*

Log (Proportion)

Standard error

**eFigure 1. Funnel Plot of the Proportion of Time in Spent in the Therapeutic Range**

Egger bias P=0.03

*-2.0*

*-1.5*

*-1.0*

*-0.5*

*0.12*

*0.09*

*0.06*

*0.03*

*0.00*

Log (Proportion)

Standard error

**eFigure 2. Funnel Plot for the Proportion of Time Spent Below the Therapeutic Range**

Egger bias P = 0.07

*-4*

*-3*

*-2*

*-1*

*0.12*

*0.09*

*0.06*

*0.03*

*0.00*

Log (Proportion)

Standard error

**eFigure 3. Funnel Plot of the Proportion Time Spent Above the Therapeutic Range**

Egger bias P=0.47

*-1.1*

*-0.9*

*-0.7*

*-0.5*

*-0.3*

*0.12*

*0.10*

*0.08*

*0.06*

*0.04*

*0.02*

*0.00*

Log (Proportion)

Standard error

**eFigure 4. Funnel Plot of the Proportion of International Normalized Ratio Measurements in the Therapeutic Range**

Egger bias P=0.96

*-3.0*

*-2.5*

*-2.0*

*-1.5*

*-1.0*

*-0.5*

*0.1125*

*0.0750*

*0.0375*

*0.0000*

Log(Proportion)

Standard error

**eFigure 5. Funnel Plot of the Proportion of International Normalized Ratio Measurements Below the Therapeutic Range**

Egger bias P=0.37

*-3.3*

*-2.3*

*-1.3*

*-0.3*

*0.1125*

*0.0750*

*0.0375*

*0.0000*

Log (Proportion)

Standard error

**eFigure 6. Funnel Plot of the Proportion of International Normalized Ratio Measurements Above the Therapeutic Range**

Eggers bias P=0.31

*0.0*

*0.3*

*0.6*

*0.9*

*0.3*

*0.2*

*0.1*

*0.0*

Proportion

Standard error

**eFigure 7. Funnel Plot of the Proportion of Thromboembolic Events that Occurred Below the Therapeutic Range (INR <2.0)**

Egger bias P=0.69

*0.05*

*0.30*

*0.55*

*0.80*

*0.25*

*0.20*

*0.15*

*0.10*

*0.05*

*0.00*

Proportion

Standard error

**eFigure 8. Funnel Plot of Proportion of Major Hemorrhagic Events that Occurred Above the Therapeutic Range (INR > 3.0)**
